# 6IMPOSE: bridging the reality gap in 6D pose estimation for robotic grasping

**DOI:** 10.3389/frobt.2023.1176492

**Published:** 2023-09-27

**Authors:** Hongpeng Cao, Lukas Dirnberger, Daniele Bernardini, Cristina Piazza, Marco Caccamo

**Affiliations:** ^1^ School of Engineering and Design, Technical University of Munich, Munich, Germany; ^2^ School of Computation, Information and Technology, Technical University of Munich, Munich, Germany; ^3^ Munich Institute of Robotics and Machine Intelligence, Technical University of Munich, Munich, Germany

**Keywords:** 6D pose estimation, RGBD image, synthetic data, robotic grasping, Sim2real

## Abstract

6D pose recognition has been a crucial factor in the success of robotic grasping, and recent deep learning based approaches have achieved remarkable results on benchmarks. However, their generalization capabilities in real-world applications remain unclear. To overcome this gap, we introduce 6IMPOSE, a novel framework for sim-to-real data generation and 6D pose estimation. 6IMPOSE consists of four modules: First, a data generation pipeline that employs the 3D software suite Blender to create synthetic RGBD image datasets with 6D pose annotations. Second, an annotated RGBD dataset of five household objects was generated using the proposed pipeline. Third, a real-time two-stage 6D pose estimation approach that integrates the object detector YOLO-V4 and a streamlined, real-time version of the 6D pose estimation algorithm PVN3D optimized for time-sensitive robotics applications. Fourth, a codebase designed to facilitate the integration of the vision system into a robotic grasping experiment. Our approach demonstrates the efficient generation of large amounts of photo-realistic RGBD images and the successful transfer of the trained inference model to robotic grasping experiments, achieving an overall success rate of 87% in grasping five different household objects from cluttered backgrounds under varying lighting conditions. This is made possible by fine-tuning data generation and domain randomization techniques and optimizing the inference pipeline, overcoming the generalization and performance shortcomings of the original PVN3D algorithm. Finally, we make the code, synthetic dataset, and all the pre-trained models available on GitHub.

## 1 Introduction

Reliable robotic grasping remains a challenge in many precision-demanding robotic applications, such as autonomous assembly (Li and Qiao, 2019) and palletizing (Lamon et al., 2020). To overcome this challenge, one approach is to accurately recognize the translation and orientation of objects, known as 6D pose, to minimize grasping uncertainty (Kleeberger et al., 2020). Recent learning-based approaches leverage deep neural networks (DNNs) to predict the 6D object pose from RGB images, achieving promising performance. Nonetheless, estimating 6D poses from RGB images is challenging. Perspective ambiguities, where the appearances of the objects are similar under different viewpoints, hampereffective learning. This problem is further exacerbated by occlusions in cluttered scenarios (Zakharov et al., 2019). Additionally, as in many computer vision tasks, the performance of the algorithms is vulnerable to environmental factors, such as lighting changes and cluttered backgrounds (Peng et al., 2019). Furthermore, using learning-based methods requires a substantial amount of annotated training data, making it a limiting factor in practical applications as data labeling is time-consuming and costly.

To address the challenges faced by RGB-based approaches, RGBD-based 6D pose estimation algorithms leverage the additional modality from depth images, where the lighting and color-independent geometric information is presented. One way to leverage depth images is to use the depth for fine pose refinement based on the coarse pose predicted from RGB images (Kehl et al., 2017; Sundermeyer et al., 2018). In this case, the initial poses are estimated from the RGB images using DNNs, and the depth information is used to optimize the pose with the Iterative Closest Point algorithm (ICP) to increase the accuracy. Another approach is to convert the depth image into point clouds, from which the 6D Pose is predicted (Chen et al., 2020; Hagelskjær and Buch, 2021). Due to the unstructured nature of the data, working directly on the point cloud is computationally expensive. Chen et al. (2020); Hagelskjær and Buch (2021) first use an instance detection network to segment the target from the RGB images and crop the point cloud correspondingly. After that, point cloud networks work on the cropped point cloud to predict the 6D pose.

As an alternative, the geometric features can be directly extracted from the point cloud using DNNs and merged with the RGB features (Li C. et al., 2018; Xu et al., 2018; Wang C. et al., 2019; He et al., 2020a; 2021; Lin et al., 2022). Typically, the extracted features of both modalities are matched geometrically and concatenated before further processing (Li C. et al., 2018; Xu et al., 2018; Wang C. et al., 2019; He et al., 2020a). This approach is simple to implement and simplifies training as the feature extraction networks can be pre-trained in isolation on the available image and point cloud data sets. However, the feature extraction on both modalities could not benefit from each other to enhance representation learning, as the feature extraction DNNs do not communicate. On the other hand, FFB6D (He et al., 2021) achieves better performance by exploring bidirectional feature fusion at different stages of feature extraction. In this way, the local and global complementary information from both modalities can be used to learn better representations. Moreover, by primarily localizing the target object and excluding the irrelevant background, the feature extraction could be more concentrated on the region of interest, thus the performance can be further improved (Lin et al., 2022).

After feature extraction, different approaches exist to derive the object pose. Direct regression uses dense neural networks to regress to the object’s pose directly (Zhou et al., 2019). While this approach allows end-to-end learning and does not require decoding the inferred pose, the optimization of the DNNs is usually difficult due to the limitation of the mathematical representation for the orientation (Zhou et al., 2019). Another common approach is the prediction of orientation-less keypoints and retrieving the pose by their geometric correspondence. He et al. (2020a, 2021); Lin et al. (2022) use DNNs to predict the keypoints in 3D space, and then compute the 6D pose via geometry matching on paired predicted keypoints and ground-truth keypoints.

State-of-the-art 6D pose estimation algorithms have achieved excellent performance as evaluated on benchmarks (Xu et al., 2018; Wang C. et al., 2019; He et al., 2020a; 2021; Chen et al., 2020; Lin et al., 2022). However, these benchmarks’ training and validation data are often correlated, as they are commonly sourced from video frames. Additionally, they may contain environmental features that can bias the learning process and simplify the inference. These factors raise concerns about the generalization of these algorithms and their ability to perform well in real-world scenarios.

Applying the state-of-the-art algorithms to practical robotic applications is non-trivial as the training of 6D pose estimation algorithms has a high demand for annotated data (Kaskman et al., 2019). 6D pose labeling of images is time and labor intensive, which limits the availability of datasets. On the other hand, using modern simulations to generate synthetic data for training DNNs shows great potential with low cost and high efficiency. For RGB-based approaches, Sundermeyer et al. (2018) and Kehl et al. (2017) render 3D meshes in OpenGL to generate synthetic RGB images with random backgrounds from commonly used computer vision datasets, for example, Pascal VOC (Everingham and Winn, 2012) or MS COCO (Lin et al., 2014). Some RGBD approaches (Wang C. et al., 2019; He et al., 2020a; He et al., 2021; Lin et al., 2022) use image composition in RGB and only render depth for the labeled objects. Recently, modern simulations, such as Unity or Blender, enable realistic rendering for full RGBD images, making these engines popular for generating high-quality training datasets (Hagelskjær and Buch, 2021).

Unfortunately, the performance of models solely trained on synthetic data often deteriorates when tested on real images due to the so-called *reality-gap* (Sundermeyer et al., 2018; Xiang et al., 2018). To mitigate the *reality-gap*, domain randomization techniques are often applied to the synthetic data (Tobin et al., 2017). Domain randomization can be applied to different aspects of image generation. Before rendering, the scene can be randomized by varying the pose of objects, backgrounds, lighting, and the environment to cover as many scenarios as possible (Liu et al., 2016; Sundermeyer et al., 2018; Hagelskjær and Buch, 2021). After rendering, the RGB and depth images can be directly altered, for example, changing image contrast, saturation or adding Gaussian blur, and color distortion (Kehl et al., 2017; Sundermeyer et al., 2018). The depth images can be randomized by injecting Gaussian and Perlin Noise (Thalhammer et al., 2019b) to approximate the noise presented on a real camera.

An alternative that recent works have explored is style transfer techniques. Rojtberg et al. (2020) introduced Style-transfer Generative Adversarial Networks (GANs) to bridge the domain gap in synthetic pose estimator training. Similarly, Ikeda et al. (2022) employed instance-level style transfer. Notably, while these style transfer techniques offer a promising direction in addressing domain adaptation challenges for 6D pose estimation, they still necessitate the use of real images, albeit in reduced quantities, to capture style and texture nuances.

Many works (Kehl et al., 2017; Sundermeyer et al., 2018; Hagelskjær and Buch, 2021) on 6D pose estimation from synthetic data only evaluate on benchmarks, however, the performance in the real world remains unclear. Zhang et al. (2022) deploy 6D pose estimation DNNs to real-world robotic grasping, showing promising performance when tested under normal lighting conditions in a structured environment. When tested in unstructured scenarios, where environmental conditions can be inconsistent, the learned algorithms often need real-world data for fine-tuning to bridge the domain gap and achieve comparable performance (Li Y. et al., 2018; Deng et al., 2020).

This work aims to enhance the reliability of robotic grasping through the use of 6D pose estimation techniques. To address the limitations of existing state-of-the-art approaches and minimize the requirement for manual data labeling, we present 6IMPOSE, a new framework for sim-to-real data generation and 6D pose estimation. The base of 6IMPOSE is the synthetic data generation pipeline that employs the 3D software suite Blender to create synthetic RGBD image datasets with 6D pose annotations. We also include an annotated RGBD dataset of five household objects generated using the proposed pipeline. The object detection module of 6IMPOSE consists of a real-time two-stage 6D pose estimation approach that integrates the object detector YOLO-V4 (Wang et al., 2021) and a streamlined, real-time version of the PVN3D 6D pose estimation algorithm (He et al., 2020a) for time-critical robotics applications. Furthermore, we provide the codebase to facilitate the integration of the vision system into a robotic grasping experiment.

We evaluate the proposed 6IMPOSE framework and 6D pose estimation algorithm on the LineMod dataset (Hinterstoisser et al., 2012). The results show a competitive performance with 83.6% pose recognition accuracy, comparable to the state-of-the-art methods. Notably the models were trained on synthetic data that was uncorrelated to the validation data. To validate the effectiveness of the proposed approach in real-world scenarios, we conducted robotic grasping experiments under varying lighting conditions. We achieved an overall success rate of 87% for five different household objects. To the best of our knowledge, this work is the first to systematically and successfully test sim-to-real 6D pose estimation in robotic grasping. As a contribution to the robotic grasping and related communities, we make the code, synthetic dataset, and all the pretrained models available on Github^1^.

This work is structured as follows: Section 1 describes the background of 6D pose estimation in robotic grasping and related work for the proposed data-generation pipeline and two-stage pose estimation algorithm. The design and implementation for the proposed approaches are introduced in detail in Section 2. The proposed approach is evaluated with the experimental setup discussed in Section 3 and results presented in Section 4. Section 5 concludes the work and gives a brief outlook on future work.

## 2 Materials and methods

In this section, we first introduce a data preparation pipeline for synthetic data generation and augmentation. Second, we present a two-stage approach to solve the 6D pose estimation problem in real time for robotic applications.

### 2.1 Synthetic data generation

In this work, the synthetic data is generated in Blender (Community, 2018) by leveraging its state-of-the-art raycasting rendering functionality. To render RGBD images, a textured 3D model of the object is required, which can be derived from CAD data or collected by 3D scanning.

#### 2.1.1 Image Generation

**FIGURE 1:**
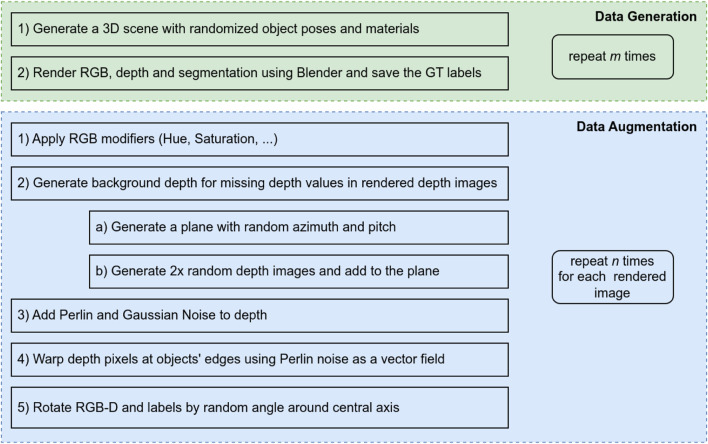
The pipeline to generate a dataset containing *m* ⋅ *n* data points is shown here. In the first generation section, the images are created using Blender. In order to the increase the size of the dataset each of the images is augmented multiple times, by the pipeline in the second section.

Image Generation Given a set of objects, we generate a separate dataset for each object of interest, with the other objects and additional unrelated objects acting as distractors. We show the order of operations to generate the final augmented datasets in Figure 1. For each scene to be rendered, we randomly place the objects in the camera’s view. In order to avoid overfitting on the color during training, we recolor 25% of the distracting objects with the dominant color of the main object. Moreover, the distractors’ optical properties, such as surface roughness and reflectivity, are varied to further increase the variety of generated images. During simulation, the randomly placed distracting objects can severely occlude the main object, which makes the main object not clearly visible, resulting in invalid training data. To avoid this, we check whether the centroid of the main object is occluded, in which case, we move the occluding objects to the back of the main object.

We sample images from SUN 2012 (Xiao et al., 2010) to use as backgrounds in Blender. Instead of adding the backgrounds to the images after rendering, we follow the idea of image-based lighting, where the background images are physically rendered as infinite spheres around the scene and emit light. Therefore, the backdrop images affect reflections and lighting conditions in the scene. Furthermore, random point lights are added to the scene with arbitrary power and position. Once the scene is constructed, Blender starts rendering to generate RGB and depth images.

#### 2.1.2 Labels

Labels The segmentation mask can be directly rendered using the object ID feature in Blender. The position and rotation of all objects and the camera is known, and the ground truth transformation matrix *Rt* can be derived accordingly. The labels for each image are then saved to separate JSON files for each image.

### 2.2 Data augmentation

After rendering the images, we apply several augmentation techniques to mitigate the *reality-gap*. Transformations that would change the object’s position in the image would invalidate the ground truth labels, except rotations around the imaging sensor’s central axis. This type of rotation preserves the object’s pose relative to the camera, allowing us to adjust the labels accordingly. By rotating each image around the central axis, we can effectively multiply our training data and apply the following techniques separately to each rotated image.

#### 2.2.1 RGB data augmentation

The synthetic RGB images are augmented by randomizing saturation, brightness, hue and contrast, sharpening and blurring. Moreover, we add Gaussian and smooth 2D Perlin noise as in Perlin (2002) to each color channel to cover different environments and sensors.

#### 2.2.2 Perlin noise

Perlin noise is a type of gradient noise developed by Ken Perlin in 1983. It’s widely used in computer graphics to generate natural-looking textures, shapes, and motion. In our work, we use Perlin noise to simulate real-world data’s random variations and inconsistencies. For example, we add Perlin noise to the depth channel to simulate the warped point clouds observed from real depth cameras, and we use it to create missing regions in the synthetic depth image, simulating the missing depth problem caused by strong reflections or limitations of the depth sensor.

#### 2.2.3 Depth data augmentation

The synthetic depth images rendered from simulations are noiseless and almost perfect. This is not the case for images obtained from a real depth camera, where the depth values are often inconsistent and incomplete (Mallick et al., 2014). To approximate inconsistent depth values, we introduce Gaussian and Perlin noise. Similar to Thalhammer et al. (2019b), pixel-level Gaussian noise is added to the synthetic depth images resembling a blurring effect. Smooth Perlin noise has been shown to significantly increase performance when learning from synthetic depth data (Thalhammer et al., 2019a). We create Perlin noise with random frequency and amplitude and add it directly to the depth channel. The introduced Perlin noise shifts each depth point along the perceived Z-axis, resulting in a warped point cloud, similar to the observed point clouds of real depth cameras.

In real RGBD images, a misalignment can be observed between depth and RGB images. Similar to Zakharov et al. (2018), we use Perlin noise again to additionally warp the depth image in the image plane. Instead of using a 3D vector field to warp the entire depth image, we restrict warping to the edges of the objects. We apply a Sobel filter to detect the edges and obtain edge masks. We then shift the pixels on the edges using a 2D vector field generated using Perlin noise.

The rendered depth images have no depth information where there is no 3D model, resulting in large empty areas between objects. However, it is also essential to simulate plausible depth values for the background (Thalhammer et al., 2019a).

The background depth is based on a randomly tilted plane, to which we add a random Gaussian noise. The noise is sampled on a grid over the image and then interpolated. Additional Gaussian noise is sampled from a second grid and again interpolated. Due to the random and independent choices of grid sizes and interpolation for the two grids, we can achieve a wide variety of depth backgrounds. By adding an appropriate offset, we guarantee that the artificial background is in close proximity to the main object; hence, making object segmentation from the background more difficult. The artificial depth background then replaces empty depth pixels in the original synthetic depth image.

In the real depth images, some regions might miss the depth values and are observed as holes due to strong reflections of the object or other limitations of the depth sensor (Mallick et al., 2014). To simulate the missing depth problem, we first generate a random 2D Perlin noise map, which is converted to a binary masking map based on a threshold. This binary masking map is then used to create missing regions in the synthetic depth image. While this method is not an accurate simulation, we found this approximation, in combination with the other augmentation strategies, helpful to improve the accuracy of the neural network.

In real-world scenarios, our model needs to handle a variety of challenges, such as changes in lighting, occlusions, variations in object appearance, and inconsistencies in depth values. The data augmentation techniques described here were chosen to simulate these conditions and improve our model’s ability to generalize to real-world data. Our experiments show that these techniques significantly improved the model’s accuracy and robustness in diverse conditions. We believe that this comprehensive data augmentation strategy is a key factor in the success of our approach.

### 2.3 A two-stage 6D pose estimation approach

**FIGURE 2:**
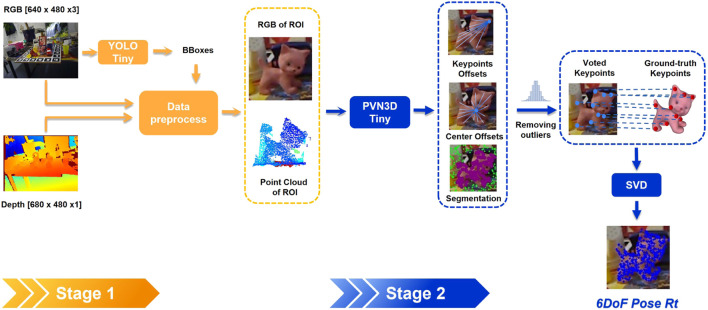
A two-stage pose estimation approach showing the object detection with YOLO-tiny to localize the object of interest at the first stage, followed by the 6D object pose estimation with PVN3D-tiny at the second stage.

The goal of 6D pose estimation is to estimate the homogeneous matrix *Rt* ∈ SE(3), which transforms the object from its coordinate system to the camera’s coordinate system. This transformation matrix consists of a rotation *R* ∈ *SO*(3) and the translation $t\in R^{3}$ of the target object. In this work, we use PVN3D (He et al., 2020a) to infer the homogeneous matrix *Rt* on the cropped region of interest (ROI) identified by a YOLO-V4-tiny (Wang et al., 2021) object detector. This two-stage approach is shown in Figure 2.

The RGB image is processed at the first stage using YOLO-V4-tiny, which provides several candidate bounding boxes and confidence scores. The bounding box with the highest confidence score for a specific object determines the ROI. Given the ROI, the cropped area is the smallest square centered on the ROI and including it, that is a multiple of the PVN3D input size (e.g., 80 × 80, 160 × 160, …). The square cropped images are then resized to 80 × 80 using nearest neighbor interpolation.

Following PVN3D (He et al., 2020a) and PointNet++ (Qi et al., 2017), the point cloud is enriched by appending point-wise *R, G, B* values and surface normals. We estimate the surface normal vectors by calculating the depth image’s gradients and the pixel-wise normals geometrically as in Holzer et al. (2012). Differently from the original PVN3D (He et al., 2020a) implementation, where the nearest neighbor approach is used to compute the normals from unstructured point clouds, calculating normals from structured depth image is more computationally efficient (Nakagawa et al., 2015). This also allows us to use a GPU-based gradient filter in TensorFlow. The resulting point cloud is then randomly subsampled to increase computational efficiency.

In the second stage, PVN3D is used for the pose estimation, with PSPNet (Zhao et al., 2017) and PointNet++ (Qi et al., 2017) as backbones to extract RGB and point cloud features separately. The extracted latent features are then fused by DenseFusionNet (Wang C. et al., 2019) at pixel level. Because of the resizing of the cropped RGB image, we map the resized features back to the nearest point in the point cloud. Shared MLPs are then used to regress to the point-wise segmentation and keypoints offsets $\left\{of_{i}\right\}\in R^{3}$ .

To obtain the final object pose, the point-wise segmentation filters out background points and the keypoint offset are added to the input point cloud to get keypoint candidates. In He et al. (2020a), keypoint candidates are clustered by using Mean-Shift clustering for the final voted keypoints $\left\{\tilde{kp_{i}}\right\}\in R^{3}$ . However, the Mean-Shift algorithm works iteratively, preventing an efficient GPU implementation with deterministic execution time. To make the keypoint voting temporally deterministic, we first select a fixed amount of point cloud points for each keypoint with the smallest predicted offset. Compared to random sampling, this selection method already removes those outliers that show a high offset. To eliminate any further outliers, we filter out any keypoint candidate whose distance to the mean prediction *μ* exceeds the standard deviation *σ*, i.e., the offsets *of*_*i*_ will be masked out if |*of*_*i*_ − *μ*| > *σ*. After removing outliers, we apply global averaging on {*x*, *y*, *z*} axis to obtain the voted keypoints $\left\{\tilde{kp_{i}}\right\}\in R^{3}$ . The *SE(3)* transformation matrix *Rt* between the predicted keypoints $\left\{\tilde{kp_{i}}\right\}$ and the reference model keypoints {*kp*_*i*_} can be found by solving the following least-squares problem:

$$\underset{R,t}{\min}\underset{i}{\sum }\|R\cdot kp_{i}+t-\tilde{kp_{i}}{\|}^{2}\;,$$ (1)

where ***R*** is the rotation matrix, and *t* is the translation vector. We use singular value decomposition (SVD) to find the optimal R* and t* that minimize Eq. 1. Specifically, given two sets of points {*kp*_*i*_} and $\left\{\tilde{kp_{i}}\right\}$ , SVD decomposes the cross-covariance matrix *H* into three different matrix as follows:

$$H=U\Sigma V^{T}\;,$$ (2)

where *U* and *V* are orthogonal matrices and **Σ** is a diagonal matrix containing the singular values of *H*. The optimal rotation matrix is calculated as: R* = *V* ⋅ *U* ^*T*^, and the translation vector *t* is obtained by subtracting the center of the reference points *kp*_*c*_ rotated with R* from predicted center point $\tilde{kp_{c}}$ , as summarized in Algorithm 1. To improve the run time performance, we implement all operations with TensorFlow and run them on the GPU.

Algorithm 1 SVD-Based Optimal Pose Estimation.

 1: **Input**: 3D model points {*kp*_*i*_}, detected object points $\left\{\tilde{kp_{i}}\right\}$

 2: **Output**: Rotation matrix *R* and translation vector *t*
 3: **function** SVD_Optimal_Pose( $\left\{kp_{i}\right\},\left\{\tilde{kp_{i}}\right\}$ )
 4:   *kp*_*c*_ ←Centroid({*kp*_*i*_})
 5: $\tilde{kp_{c}}\leftarrow \text{Centroid}\left(\left\{\tilde{kp_{i}}\right\}\right)$

 6:   {*kp*_*i*_}←{*kp*_*i*_} − *kp*_*c*_     ⊳ Translate both sets of points to the origin
 7: $\left\{\tilde{kp_{i}}\right\}\leftarrow \left\{\tilde{kp_{i}}\right\}-\tilde{kp_{c}}$

 8: $H\leftarrow \left\{kp_{i}\right\}\cdot {\left\{\tilde{kp_{i}}\right\}}^{T}$ ⊳ Compute cross-covariance matrix
 9:   *U*, Σ, *V* ^*T*^ ←SVD(*H*)     ⊳ Compute SVD of *H*
 10:   R* ← *V* ⋅ *U* ^*T*^     ⊳ Compute rotation matrix
 11: $t^{*}\leftarrow \tilde{kp_{c}}-R^{*}\cdot kp_{c}$ ⊳ Compute translation vector
 12:   **Return** *R*, *t*
 13: **end function**

In this two-stage pose estimation pipeline, the pose estimation accuracy is improved by cropping the image to the ROI, as only the relevant part of the data is processed. With the same number of sampling points, the sampled point cloud from the cropped image is denser, providing PointNet++ with richer geometric information for feature processing, which can also be observed in Lin et al. (2022). Given the cropped input, we could build the PVN3D with only about 8 million parameters, which is approximately 15% of the original implementation (He et al., 2020b). In our test on the LineMOD dataset, the reduced PVN3D performs similarly to the original model. We refer to the reduced PVN3D model as PVN3D-tiny.

## 3 Experiments

In this section, we study the effectiveness of the proposed synthetic data preparation pipeline and the two-stage 6D pose estimation algorithm. Specifically, we use 3D models of objects to generate synthetic RGBD data for training the proposed algorithm. After training, we deploy the trained deep models for 6D pose estimation on LinemMod dataset to study ADD(S) pose estimation accuracy and in a real robotic grasping experiment to study grasping success rate (SR).

### 3.1 Synthetic data preparation

In this work, we are interested in the 6D pose estimation of a single object in cluttered environments. For each object, we render scenes in Blender to generate 20 k synthetic images using the provided 3D model. We then augment the rendered dataset by rotating each image around the center of the image 16 times, resulting in around 300 k images. We discard the images where the object is out of view and pad the images with zeros for empty areas after rotation. Additionally, each image is augmented offline by applying the domain randomization techniques introduced in Section 2. To train PVN3D-tiny, we crop the RGBD to obtain the region of interest according to the ground truth bounding boxes. Furthermore, we generate the point-wise semantic masks and keypoints offsets from the cropped RGBD images using the ground truth poses and segmentation masks.

### 3.2 Synthetic data inspection

**FIGURE 3:**
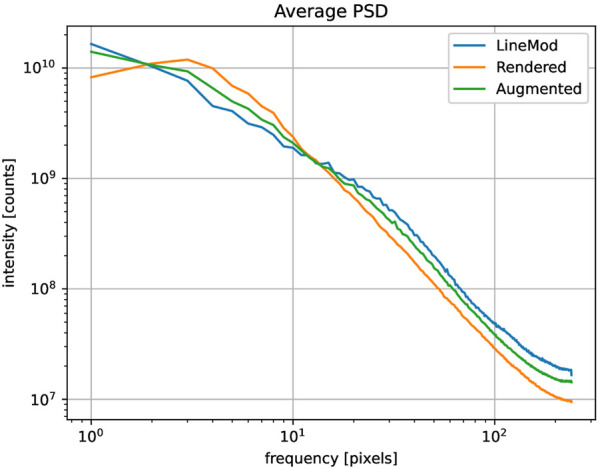
The plot showing the qualitative average power spectral density (PSD) of depth images with respect to frequencies for the object “cat” from LM dataset over 50 randomly sampled images.

To quantify the *reality-gap* we sample 50 RGBD images from the synthetic and real datasets and compare global statistics. For RGB images, we compute the average and the standard deviation for brightness and saturation, as we qualitatively observed that these two factors strongly influence the appearance of the generated data. By comparing the statistic of brightness on the synthetic and real subsets, we can optimize the average power and randomization of the point lights and the light-emitting background in Blender. Similarly, with the statistics of saturation, we can optimize the color management in the Blender. To study the statistics of depth images, we use the average power spectral density (PSD) and compare the average distribution on frequencies, as shown in Figure 3. Studying PSD on frequencies allows us to inspect the structures of the depth images and adjust accordingly the frequency of the Perlin noise used for depth augmentation. It can be seen that the augmented depth images are closer in frequency distribution to the real images than the non-augmented ones. This indicates that depth augmentation reduces the gap between the synthetic and real data.

**FIGURE 4:**
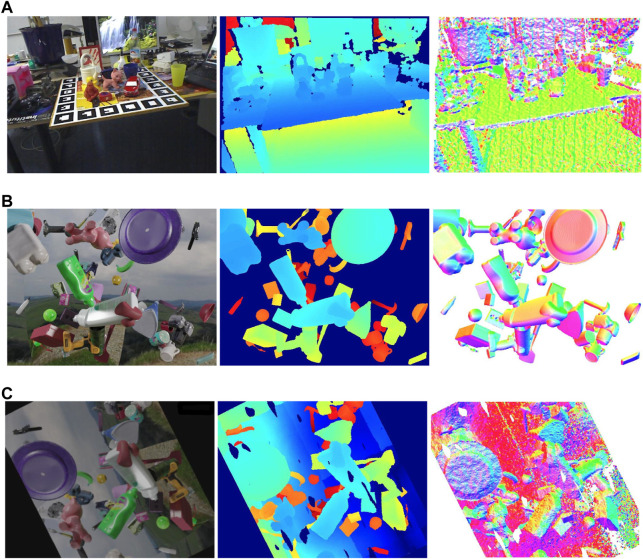
The figure showing the visualization of RGB images, depth images and surface normals from the left to the right. **(A)** Real data. **(B)** Rendered synthetic data. **(C)** Augmented synthetic data.

Examining global statistics for RGBD images is efficient, as it does not require real annotations. This examination also enables us to identify the “reality gap” qualitatively and adjust the data generation parameters, such as brightness and depth frequencies, to align the synthetic data closer to the real data. Figure 4 illustrates examples of real and synthetic images.

### 3.3 Implementation

The synthetic data generation pipeline is implemented in Python using Blender’s API. The data randomization and preprocessing are implemented using TensorFlow, accelerating the processing with GPUs. As for the two-stage 6D pose estimation approach, we use the original Darknet implementation (Wang et al., 2021) of YOLO-V4-tiny for the object detection at the first stage and PVN3D-tiny, implemented in TensorFlow, in the second stage.

### 3.4 Training and evaluation on LineMod dataset

To address single object 6D pose estimation problem on LineMod, we separately train a binary YOLO-V4-tiny model and a PVN3D-tiny model for each object of the LineMOD dataset. The YOLO-V4-tiny model is trained using the Darknet framework (Redmon, 2016), and PVN3D-tiny is trained in TensorFlow (Abadi et al., 2016). All deep neural networks are trained from scratch using only synthetic data without any pretrained models. After training, we build the two-stage 6D pose estimation pipeline by combining YOLO-V4 and PVN3D. We follow He et al. (2020a) to evaluate the 6D pose estimation performance on the annotated real images provided in LineMOD. The 6D pose estimation performance is measured using ADD(S) metrics (Hinterstoisser et al., 2012). ADD measures the average distance between the ground truth point cloud and the point cloud transformed with predicted *R*, *t*, which can be defined as follows:

$$ADD=\frac{1}{m}\underset{v\in O}{\sum }\|\left(Rv+t\right)-\left(R^{*}v+t^{*}\right)\|,$$ (3)

where *m* is the number of the sampled points, R*, t* is the ground truth pose, and *v* ∈ *R* ^3^ denotes a vertex from the object O . Similarly, the ADDS metric measures the average minimum distance between two point clouds as:

$$ADDS=\frac{1}{m}\underset{v1\in O}{\sum }\underset{v2\in O}{\min}\|\left(Rv+t\right)-\left(R^{*}v+t^{*}\right)\|,$$ (4)

Compared to ADD, ADDS measures the distance to the nearest point instead of correspondent mesh points. ADDS is better suited for symmetrical objects because ADD yields low scores if the object’s pose is different from the ground truth, even if the pose corresponds to an invariant rotation. The success rates on test images are used to quantify the pose estimation performance. A threshold of 10% of the object’s diameter is typically used to classify a prediction as successful.

### 3.5 Robotic grasping

**FIGURE 5:**
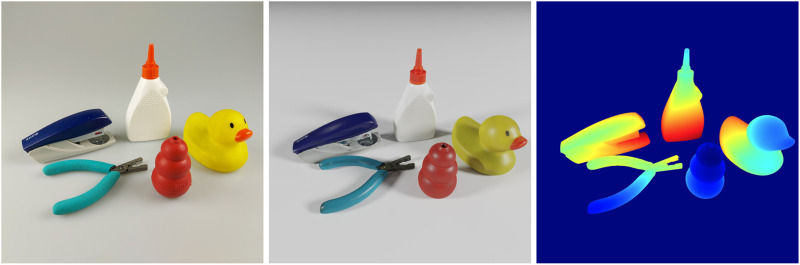
The figure showing the photographed, photo-realistically rendered RGB and rendered depth images from the left to the right for the five selected household objects.

**FIGURE 6:**
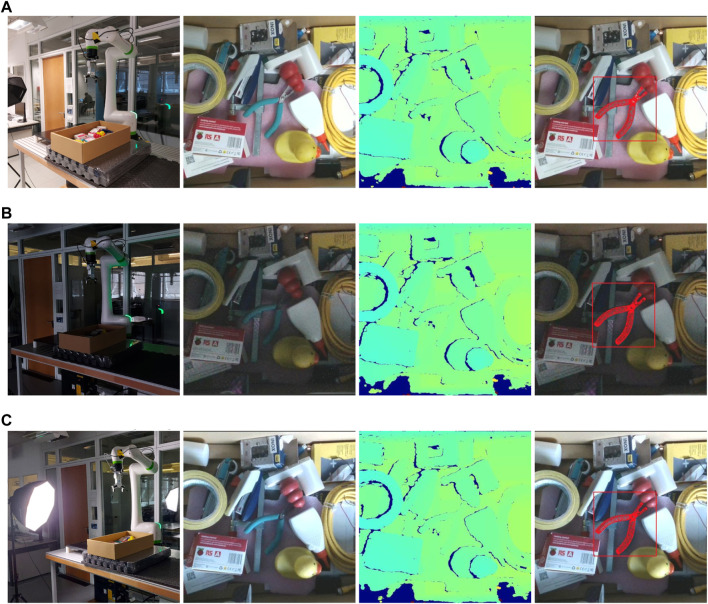
The figure showing the real world grasping experiments under varying lighting conditions with the experimental setup, RGB view, depth view and the predicted pose of pliers on RGB shown from the left to the right. **(A)** Diffused lighting conditions. **(B)** Low lighting conditions. **(C)** Spot lighting conditions.

We train the proposed approach for pose estimation from purely synthetic images, to perform robotic grasping experiments. We choose five household objects: a rubber duck, a stapler, a chew toy for dogs, a glue bottle, and pliers, as shown in Figure 5, for which the 3D models of the objects are obtained using a Shining3D Transcan C 3D scanner. We generate synthetic training data and train a multi-classes detector YOLO-V4 to localize the target object and train multiple PVN3D-tiny models to estimate the poses of different target objects, as described in Section 2. For the grasping experiments, we use a robotic manipulator Fanuc CRX 10iAL with a custom Python interface. As endeffector, we use an OnRobot RG2 gripper. Attached to the endeffector is an Intel Realsense D415 which is used to obtain the RGBD images. This setup is then used to perform 50 grasp attempts per object in three different lighting conditions, which yields 750 grasps in total. The three lighting conditions are diffused, low and spot lighting, to test the algorithm’s robustness to different lighting levels, as shown in Figure 6.

#### 3.5.1 Grasping strategy

The following approach is used to conduct grasping experiments. The robot starts by moving to a predefined home position where the entire bin is visible in the camera’s field of view. The object of interest is then identified using YOLO and PVN3D-tiny. To ensure that possible collisions around the object can be observed, the robot moves its end-effector directly above the object. This is important when the object is close to the edge of the camera’s view and surrounding obstacles may be out of sight. A safe grasp pose is selected using the pose estimation and grasp selection method. A smooth and tangential trajectory, using a Bézier curve, is generated to approach the object. The gripper is closed when it reaches the grasp pose and the object is lifted out of the bin. To conclude one grasping attempt, the object is dropped back into the bin after the robot returns to the initial home position. If the object can be grasped and lifted without slipping, this grasp is regarded as a success, a failure otherwise. We distinguish the failure cases between a missed grasp and a collision, to identify the cause of failure, which can be the pose estimation or the collision avoidance.

#### 3.5.2 Grasp pose estimation

Typically, grasp pose estimation follows either an algorithmic or data-driven approach (Eppner et al., 2021; 2022). In this paper, we leverage a simple algorithmic approach similar to Kleeberger et al. (2022). Local grasp poses in the object’s coordinate frame are generated offline and beforehand. With the estimated pose of the object, the local grasp pose can be lifted to the global coordinate frame as a target pose for the robotic manipulator. Generally, it is not required to find all grasp poses or the best one, but to find a set of poses, that cover most directions from which the robot may approach the object. Therefore, a list of grasp candidates is generated, that will enable the robotic gripper to securely grasp the object.

We use a sampling based grasp pose estimation using the available mesh of the objects, where randomly sampled points on the objects’ surface are considered as possible contact points. For each connecting line between a pair of points, we generate 24 grasp poses rotated around the connecting line. Additionally we generate the corresponding antipodal grasps. A grasp pose is considered valid if it meets following criteria.

• The surface curvature on the mesh should not prevent a stable friction grasp. Therefore, no sharp edges or concave surfaces are considered;
• The contact surfaces should be perpendicular to the connecting line. This ensures a stable friction grasp;
• The gripper bounding box should not collide with the object.

**FIGURE 7:**
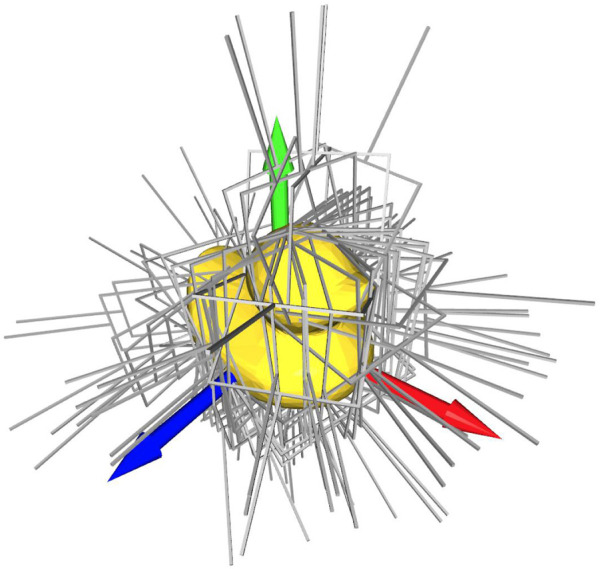
The figure showing the automatically generated grasp poses for the household object “duck”.

The remaining grasps are then downsampled, using sparse anchor points in three-dimensional space. Our approach typically yields less than 100 grasps for each object, while still providing a high degree of coverage of all possible angles, as can be seen in Figure 7.

#### 3.5.3 Grasp pose selection

 The optimal grasp pose for an object of interest is then selected utilizing the predicted 6D pose and the pointcloud data from an RGBD camera. We first filter out grasp poses that would require the robot to approach the object from a vastly different angle than the current pose of the gripper. We then evaluate the remaining grasp poses for collisions by considering every point in the pointcloud not belonging to the object as an obstacle. The grasp poses that would lead to a collision with the obstacle points are rejected. Finally, we choose the grasp pose that maximizes the distance to the pointcloud for safety.

## 4 Results

In this section, we report the ADD(S) accuracy performance of the proposed two-stage 6D pose estimation algorithm on the LineMOD dataset after training on the synthetic data. We also report the success rate (SR) for grasping different household objects in robotic grasping experiments.

### 4.1 6D pose estimation accuracy

We evaluate the performance of the proposed 6D pose estimation approach on all objects from the LineMOD dataset. The results, alongside comparisons to state-of-the-art methods, are presented in Table 1. The performance metrics for PointFusion are sourced from Lin et al. (2022), while those for SSD-6D (Kehl et al., 2017) are taken from Sundermeyer et al. (2018). When compared to other synthetic-only trained methods, our approach achieves competitive performance with overall 83.6% pose recognition accuracy without the need for pose refinement. It performs particularly well on small objects like “ape”, “duck”, on which the SSD-6D (Kehl et al., 2017) and AAE (Sundermeyer et al., 2018) are less accurate. However, our model’s performance is suboptimal for objects like “holepuncher” and “camera”. This discrepancy might be attributed to the lower-quality textures of the LineMOD models. Being trained end-to-end on RGBD data, our approach could be more sensitive to less-detailed textures than refinement-based approaches.

**TABLE 1:** The performance of 6D pose estimation on LineMOD compares to the state-of-the-art using RGBD. The bold objects are symmetric.

	Real data						Synthetic data			
	PointFusion^1^	DenseFusion^2^*	G2L-Net^3^	PVN3D^4^	FFB6D^5^	E2EK^6^	AAE^7^*	SSD-6D^8^*	DGCNN^9^*	Ours
Ape	70.4	92.3	96.8	97.3	98.4	98.7	20.55	65	97.7	78
Benchvise	80.7	93.2	96.1	99.7	100	100	64.25	80	99.8	92
Camera	60.8	94.4	98.2	99.6	99.9	99.9	63.20	78	98.3	66
Can	61.1	93.1	98	99.5	99.8	100	76.09	86	98.8	95
Cat	79.1	96.5	99.2	99.8	99.9	100	72.01	70	99.9	97
Driller	47.3	87	99.8	99.3	100	100	41.58	73	99.2	91
Duck	63	92.3	97.7	98.2	98.4	99.4	32.38	66	97.8	89
Eggbox	99.9	99.8	100	99.8	100	100	98.64	100	97.7	91
Glue	99.3	100	100	100	100	100	96.39	100	98.9	73
Holepuncher	71.8	92.1	99	99.9	99.8	100	49.88	49	94.1	61
Iron	83.2	97	99.3	99.7	99.9	100	63.11	78	100	94
Lamp	62.3	95.3	99.5	99.8	99.9	99.9	91.69	73	92.8	87
Phone	78.8	92.8	98.9	99.5	99.7	100	70.96	79	99.1	74
All	73.7	94.3	98.7	99.4	99.7	99.8	64.67	79	98.0	83.6
Speed(s)	—	0.06	0.044	0.19	0.075	0.068	—	0.1	1.0	0.046

* With refinement.

^1^ Xu et al. (2018).

^2^ Wang et al. (2019a).

^3^ Chen et al. (2020).

^4^ He et al. (2020a).

^5^ He et al. (2021).

^6^ Lin et al. (2022).

^7^ Sundermeyer et al. (2018).

^8^ Kehl et al. (2017).

^9^ Hagelskjær and Buch (2021).

In comparison to related works that solely rely on synthetic data for training, our method surpasses both AAE (Sundermeyer et al., 2018) and SSD-6D (Kehl et al., 2017). Additionally, the 6D pose estimation algorithm proposed by Hagelskjær and Buch (2021), which is based on DGCNN (Wang Y. et al., 2019), achieves an impressive average accuracy of 98%. However, it is heavily dependent on pose refinement and requires approximately one second to detect a single object.

As illustrated in Table 1, algorithms trained on real data typically outshine those trained exclusively on synthetic data. Yet, it’s worth noting that our method can attain an accuracy of roughly 94% without refinement when the ground truth bounding box is utilized for target object localization, as depicted in Table 2. This performance is on par with state-of-the-art techniques that are trained using real data. One avenue for enhancing object detection performance could be the incorporation of RGBD images (Gupta et al., 2014). In this setup, the object detector would benefit from the robust features derived from both the appearance characteristics of RGB images and the geometric attributes of depth images. This performance disparity might also be reduced by fine-tuning the object detector using a small set of annotated real data. Importantly, we did not detect any performance degradation of the object detector on our robotic grasping dataset.

**TABLE 2:** 6D pose estimation ADD(S) scores, using predicted or ground truth bounding boxes.

	Ape	Benchvise	Camera	Can	Cat	Driller	Duck	Eggbox	Glue	Holepuncher	Iron	Lamp	Phone	All
Predicted bboxes	78	92	66	95	97	91	89	91	73	61	94	87	74	83.6
GT bboxes	81	99	98	96	97	99	94	99	99	78	96	94	96	94.3

### 4.2 Run time

The efficiency of the proposed approach was evaluated on a workstation equipped with two Xeon Silver-CPU (2.1 GHz) and an NVIDIA Quadro RTX 8000 graphics card. The results, as reported in Table 3, indicate that the inference of PVN3D-tiny consumes the majority of the running time, while the other procedures have, in aggregate, similar computational requirements. For an input of 480 × 640 RGB and depth images, the proposed approach has an average running time of 46 ms per single object pose estimation. This performance is comparable **to** or better than the existing state-of-the-art methods listed in Table 1, and suitable for real-time robotic tasks. As demonstrated in the following section, the accuracy of the proposed approach is sufficient for grasping tasks.

**TABLE 3:** Running time analysis of the proposed two-stage pose estimation approach.

Procedures	Speed Mean/Std(ms)	Percent (%)
YOLO-V4 tiny	6.7/0.5	15
Pcld preproc.	8.2/5.9	18
PVN3D tiny	23.7/0.9	51
Pose regression	7.2/0.7	16
All	45.8/6.2	100

### 4.3 6DoF pose estimation in robotic applications

As shown in Table 4, the robotic arm has achieved an approximate 87% success rate (SR). The three scenarios show similar success rates, showing the algorithm’s robustness to different lighting levels. Notably, the proposed algorithm works well in low-lighting conditions. The reason could be attributed to two factors: first, the training on domain-randomized synthetic data makes the algorithm learn more robust features. Second, the depth information remains consistent under different lighting conditions, as shown in Figure 6. So the algorithm can extract sufficient features from depth to compensate for the underexposed color camera.

**TABLE 4:** Single Object grasping experiments under variable lighting conditions.

Conditions	Category	Duck	Stapler	Glue	Chewtoy*	Pliers	All
Diffused	Success	47	46	46	45	40	224
	Grasp missed	2	3	4	1	5	15
	Collision	1	1	0	4	5	11
	SR	94.0%	92.0%	92.0%	90.0%	80.0%	89.6%
	SR w.o collision	95.9%	93.9%	92.0%	97.8%	88.9%	93.7%
Low	SR	47	38	43	42	41	211
	Grasp missed	3	8	4	2	7	24
	Collision	0	4	3	6	2	15
	SR	94.0%	76.0%	86.0%	84.0%	82.0%	84.4%
	SR w.o collision	94.0%	82.6%	91.5%	95.5%	85.4%	89.8%
Spot	Success	48	43	44	39	44	218
	Grasp missed	2	2	2	1	3	10
	Collision	0	5	4	10	3	22
	SR	96.0%	86.0%	88.0%	78.0%	88.0%	87.2%
	SR w.o collision	96.0%	95.6%	95.7%	97.5%	93.6%	95.6%
All conditions	SR	94.7%	84.7%	88.7%	84.0%	83.3%	87.06%
	SR w.o collision	95.3%	90.7%	93.0%	96.9%	89.3%	93%

* symmetrical object.

In general, collision avoidance is not the focus of this research and the results regarding the accuracy of the pose estimation pipeline are more relevant. Thus it is interesting to analyse the grasping success and pose estimation failures excluding all the collision events. The collision cases are mainly due to insufficient collision checking. If we neglect the collision cases, the failure cases decrease by 50%, and the overall grasping performance achieves 93%. This suggests that a more sophisticated collision checking and grasp pose selection strategy is required and it will be the subject of future work.

#### 4.3.1 Rubber Duck

The grasping of the rubber duck is the most robust and successful of all objects. The non-regular shape of the duck with no rotational symmetries are robust features, resulting in an accurate pose estimation. Additionally, the rubber material and soft structure facilitate robotic grasping, where slight inaccuracies still lead to successful grasps.

#### 4.3.2 Glue bottle

The glue bottle achieves a good SR as well due to its shape and material, which are forgiving, similarly to those of the rubber duck. Additionally, the bright color of the glue bottle might aid in low light environments, making the object easily visible.

#### 4.3.3 Stapler

The grasping of the stapler is highly affected by the lighting conditions, with 86.0% SR under spot light and 76.0% under low light. Possibly, the stapler, due to its dark color, loses more details in low light conditions, making 6D pose estimation more difficult without properly distinguishable features.

#### 4.3.4 Chewtoy

During grasping of the chew toy, collisions have been the primary cause of failure due to its small size, as it easily gets stuck in small cavities between other objects. We observed that this is especially relevant for the chewtoy, because the round shape makes the object roll in the bin, until it gets stopped by other objects. Therefore, the primary reason for failure is not the pose estimation, but the inferior collision avoidance. Moreover under spot lighting conditions, the chewtoy is under-exposed, particularly when stuck in a hole and this makes 6D pose estimation challenging. Combined with the proximity to other objects, this leads to an increased rate of collisions.

#### 4.3.5 Pliers

In the case of grasping the pliers, we observe a higher number of missed grasps, due to the small size of the grasp handles. The grasp generation places all the grasps on the handles and, while according to the ADD most of the proposed grasp would be successful, in practice, some fail in the robotic experiment.

## 5 Conclusion and future work

This work introduces 6IMPOSE, a novel framework for sim-to-real data generation and 6D pose estimation. The framework consists of a data generation pipeline that leverages the 3D suite Blender to produce synthetic RGBD image datasets, a real-time two-stage 6D pose estimation approach integrating YOLO-V4-tiny Wang et al. (2021) and a real-time version of PVN3D He et al. (2020a), and a code base for integration into a robotic grasping experiment.

The results of evaluating the 6IMPOSE framework on the LineMod dataset Hinterstoisser et al. (2012) showed competitive performance with 83.6% pose recognition accuracy, outperforming or matching state-of-the-art methods. Furthermore, the real-world robotic grasping experiment demonstrated the robustness of the 6IMPOSE framework, achieving an 87% success rate for grasping five different household objects from cluttered backgrounds under varying lighting conditions.

The contribution of 6IMPOSE lies in its efficient generation of large amounts of photo-realistic RGBD images and successful transfer of the trained inference model to real-world robotic grasping experiments. To the best of our knowledge, this is the first time a sim-to-real 6D pose estimation approach has been systematically and successfully tested in robotic grasping.

In future work, there is potential to further improve 6IMPOSE by exploring improvements to the perception pipeline, such as using a more sophisticated pose detection network or multi-frame detection; improving the scalability and quality of the data generation process; and improving the robotic integration, for example, in areas such as collision detection, grasping pose selection, and support for more scenarios like bin picking where there are multiple instances of the same object class.

## Data Availability

The datasets presented in this study can be found in online repositories. The names of the repository/repositories and accession number(s) can be found below: https://github.com/HP-CAO/6IMPOSE.

## Footnotes

1
https://github.com/HP-CAO/6IMPOSE
